# Consensus document for lipid profile testing and reporting in Spanish clinical laboratories: what parameters should a basic lipid profile include?

**DOI:** 10.1515/almed-2023-0047

**Published:** 2023-06-05

**Authors:** Teresa Arrobas Velilla, Carlos Guijarro, Raquel Campuzano Ruiz, Manuel Rodríguez Piñero, José Francisco Valderrama Marcos, Antonio Pérez Pérez, Antonio M. Botana López, Ana Morais López, José Antonio García Donaire, Juan Carlos Obaya, Luis Castilla-Guerra, Vicente Pallares Carratalá, Isabel Egocheaga Cabello, Mercedes Salgueira Lazo, María Mar Castellanos Rodrigo, José María Mostaza Prieto, Juan José Gómez Doblas, Antonio Buño Soto, on behalf of the Multidisciplinary Task Force for Lipids and Vascular Risk

**Affiliations:** Spanish Society of Laboratory Medicine (SEQCML), Laboratory of Clinical Biochemistry, Virgen Macarena University Hospital, Seville, Spain; Spanish Society of Arteriosclerosis (SEA), Unit of Internal Medicine, Hospital Alcorcón Foundation University Hospital, Rey Juan Carlos University, Madrid, Spain; Spanish Society of Cardiology (SEC), Unit of Cardiology, Alcorcón Foundation University Hospital, Association for Vascular Risk and Cardiac Rehabilitation of the Spanish Society of Cardiology, Madrid, Spain; Spanish Society of Angiology and Vascular Surgery (SEACV), Cross-center Cádiz-Jerez Unit of Angiology and Vascular Surgery, Puerta del Mar University Hospital, Cádiz, Spain; Spanish Society of Endocrinology and Nutrition (SEEN), Section of Endocrinology, Lucus Augusti University Hospital, Lugo, Spain; Spanish Society of Gastroenterology, Paediatric Hepatology and Nutrition (SEGHNP), Unit of Paediatric Nutrition and Metabolic Diseases, La Paz University Hospital, Madrid, Spain; Spanish Society of Hypertension, Spanish League for the Fight Against Arterial Hypertension (SEH-LELHA), Unit of Arterial Hypertension, Hospital Clínico Universitario San Carlos, Madrid, Spain; Spanish Society of Family and Community Family (SEMFyC), CS La Chopera, Alcobendas, Madrid, Spain; Spanish Society of Internal Medicine (SEMI), Unit of Hypertension, Lipids and Vascular Risk, Service of Internal Medicine, Seville, Spain; Hospital Virgen Macarena, PCDV Departamento de Medicina, University of Seville, Sevilla, Spain; Spanish Society of Primary Care Physicians (SEMERGEN), Unit of Health Surveillance, Unión de Mutuas, Department of Medicine, Universitat Jaume I, Castellón, Castellón, Spain; Spanish Society of General and Family Doctors (SEMG), Family and Community Medicine, Centro de Salud Isla de Oza, Servicio Madrileño de Salud, Madrid, Spain; Spanish Society of Nephrology (SEN), Unit of Nephrology, Virgen Macarena University Hospital, Seville, Spain; Spanish Society of Neurology (SEN), Service of Neurology, Complejo Hospitalario Universitario A Coruña/Instituto de Investigación Biomédica A Coruña, Coruña, Spain; Spanish Society of Arteriosclerosis (SEA), Service of Internal Medicine, Hospital La Paz-Carlos III, Madrid, Spain; Spanish Society of Cardiology (SEC), Service of Cardiology, Virgen de la Victoria University Hospital, Málaga, Spain; Spanish Society of Laboratory Medicine (SEQCML), Service of Clinical Biochemistry, La Paz University Hospital, Madrid, Spain; Spanish Society of Diabetes (SED), Endocrinology and Nutrition Department, Hospital de la Santa Creu i Sant Pau, Barcelona, Spain; Spanish Society of Cardiovascular and Endovascular Surgery, Cardiovascular Surgery (SECCE), Regional University Hospital of Malaga, Málaga, Spain

**Keywords:** apolipoprotein B, biochemistry, cardiovascular diseases, cholesterol, consensus, lipoprotein (a)

## Abstract

Cardiovascular diseases (CVD) continue to be the main cause of death in our country. Adequate control of lipid metabolism disorders is a key challenge in cardiovascular prevention that is far from being achieved in real clinical practice. There is a great heterogeneity in the reports of lipid metabolism from Spanish clinical laboratories, which may contribute to its poor control. For this reason, a working group of the main scientific societies involved in the care of patients at vascular risk, has prepared this document with a consensus proposal on the determination of the basic lipid profile in cardiovascular prevention, recommendations for its realization and unification of criteria to incorporate the lipid control goals appropriate to the vascular risk of the patients in the laboratory reports.

## Introduction

Cardiovascular diseases (CVDs), including coronary heart disease and cerebrovascular disease are the leading cause of mortality and disability in the world [1]. In Spain, CVDs continue to be the leading cause of death, followed by tumors and COVID-19 even during the height of the pandemic [2]. As the underlying pathological process in most CVDs, arteriosclerosis is a gradual process that occurs over decades. The main associated risk factors are widely known. Among them, dislipidaemia is a well-known risk factor which control has proven to reduce cardiovascular morbimortality [3, 4]. While there is a large therapeutic armamentarium for dyslipidaemia, the level of control over lipid abnormalities is clearly suboptimal, particularly in patients with a (very) high cardiovascular risk, in whom reducing absolute risk is crucial [5], [6], [7], [8].

An update of ESC Guidelines on Cardiovascular Disease Prevention in Clinical Practice was recently published [9]. These guidelines are supported by the major Spanish scientific societies involved in cardiovascular disease, including the CEIPV (Comité Español Interdisciplinario de Prevención Vascular) [10], [11], [12], [13].

Therapeutic targets for lipid-lowering therapies have been established and widely accepted. However, the reference values provided on laboratory biochemistry reports continue to be based on the distribution of values in the general population. Unfortunately, the “desirable” values according to the level of cardiovascular risk of the patient are all too often not provided. In spite of SEA (Spanish Society of Arteriosclerosis) and 2018 SEC (Spanish Society of Cardiology) recommendations [14, 15], lipid values largely exceeding “desirable” values in terms of cardiovascular prevention [16] are often reported as “normal”, whereas “desirable” values are reported as “abnormally low”. This information may be misleading and result in therapeutic abstention in patients with “normal” values, and dose reduction in patients with “abnormally low” values. For this reason, a working group of the main scientific societies involved in the care of patients with vascular risk have prepared this document, which includes a basic consensus proposal for the determination of the basic lipid profile in cardiovascular disease prevention, a set of recommendations for its implementation, and some standard criteria to incorporate lipid control targets adjusted to the vascular risk of the patient on laboratory reports.

## Pre-analytical considerations

### How, when and in which cases should lipid profile testing be ordered?

Lipid profile is necessary to assess the risk of developing a cardiovascular disease in apparently-healthy subjects and in high-risk clinical conditions, including candidates to cardiovascular surgery. Lipid profile is also required to assess the therapeutic effectiveness of and adherence to lipid-lowering treatments. It is essential in the prevention of CVDs, especially in subjects with a high cardiovascular risk or having relatives with a high cardiovascular risk. Likewise, it is part of the global assessment of other disorders causing secondary dyslipidaemias. Our Task Force deems that recent recommendations from the Spanish Society of Cardiology [9], recently translated [10], and supported by the Spanish Committee of Vascular Prevention [13], provide appropriate reference values (Table 1A and B).

**Table 1A:** Lipid determination for assessing cardiovascular risk [17].

Patients not receiving with lipid-lowering drugs.
(1). Routine vascular risk assessment is recommended in patients presenting a major cardiovascular risk factor (i.e. familial history of early CVD, familial hypercholesterolemia, or factors such as tobacco use, arterial hypertension, diabetes mellitus, hyperlipemia, chronic renal disease, obesity or comorbidities that increase the risk for CVD). (2). Consider routine or opportunistic assessment of CVR in men >40 years and women>50 years or postmenopausal women of the general population without CV risk factors. (3). Consider follow-up assessment at 5 years (or earlier if the risk is close to therapeutic thresholds) in all subjects who have undergone screening for CVD risk during an opportunistic screening. (4). Routine CVR assessment is not recommended in men <40 years and women <50 years without known CVR factors.

**Monitoring of therapeutic efficacy and adherence to lipid lowering treatment**

(1). Before lipid-lowering therapy is initiated perform 2 determinations within a 1–2 week interval, except in case of cardiovascular event and in patient with very high risk with indication for immediate treatment. (2). Once lipid-lowering treatment has been initiated, repeat laboratory analysis. a. After an acute atherosclerotic vascular event, at 4–6 weeks. b. In patients who are stable in cardiovascular terms, at 8±4 weeks until targets are achieved. (3). Once the patient has reached the optimal lipid target, how often should lipids be measured? On a yearly basis

**Table 1B:** Lipid targets according to cardiovascular risk [9].

In cases of **very high** CVR, a 50 % reduction of the baseline value is recommended, along with and a target LDL chol <1.4 mmol/L (<55 mg/dL), non-HDL ch. <85 mg/dl and ApoB <65 mg/dL. In case of **high** CVR, a 50 % reduction of the baseline value is recommended, along with a target LDL chol <1.8 mmol/L(<70 mg/dL), non-HDL ch. <100 mg/dl and ApoB <80 mg/dL. In case of **moderate** CVR, a target LDL chol <2.6 mmol/L(<100 mg/dL), non-HDL ch. <131 mg/dl and ApoB <100 mg/dL is recommended. In case of **low** CVR, consider a target LDL <3.0 mmol/L (<116 mg/dl).
**Suspect for familial hypercholesterolemia** in patients who developed arteriosclerotic cardiovascular disease before 55 years (men) or 60 years of age (women); subjects with a relative who had early CVD; subjects with relatives with tendon xamtomata; patients with very elevated LDL chol. (Adults, > 5 mmol/L [190 mg/dL]; children>4 mmol/L [150 mg/dL]) and first-degree relatives with familial hypercholesterolemia.
In children, perform testing from 5 years of age or younger, upon suspicion for homozygous familial hypercholesterolemia (HFHo).

CVD, cardiovascular disease; CVR, cardiovascular risk; LDL-c, cholesterol associated to low density lipoproteins; Apo B, apolipoprotein B.

### Factors influencing lipid profile determination

Laboratory parameters may be influenced by multiple factors. Thus, samples should be collected when the patient is in a “stable metabolic status” [18].

**Table:** 

Recommendation 1: Testing lipid profile is not recommended in a context of non-cardiovascular acute inflammatory process. Lipid profile should be determined within the first 24 h after an acute arteriosclerotic ischemic event.

**Lifestyle and pathophysiological status of the patient:**

a) The patient should maintain regular habits in the two previous weeks prior to the blood test.
b) The patient should not do strenuous exercise before a blood test.
c) The patient should remain in sitting position 15 min before the blood test.
d) Phlebotomy should be standardised: Venous blood should be drawn with the patient in sitting position (levels of Tg and LDL-c may be lower in supine position).
e) Exclude secondary dyslipidaemias and dyslipidaemias related to drug therapy. Annex 1 [19, 20].
f) In case of an acute inflammatory process, phlebotomy should be performed at least 2–4 weeks after the process, since the process may cause a decrease in total cholesterol, LDL cholesterol, and HDL cholesterol, and an increase in triglycerides [21–24].
g) In case of acute coronary syndrome (or other acute ischemic atherosclerotic event), lipid profile should be obtained within the first 24 h [25–27]. If it is performed>24 h after the acute process, levels of total cholesterol and LDL-c may be reduced with respect to the values normally found in the patient, a phenomenon that should be taken into account in clinical decision-making. In patients that have never undergone a lipid profile test, Lp (a) testing is recommended. Although Lp (a) values may increase in acute processes, variation is modest [28, 29], which enables the detection of patients with significantly elevated Lp (a) in early stages.

**Is it necessary that lipid profile is tested in fasting conditions?**

– Most lipid parameters offer little variation, regardless of the patient being in fasting conditions or not [30].
– The main clinical guidelines do not require fasting lipid profile, at least for an initial cardiovascular risk assessment or for diagnosis of isolated hypercholesterolemia (familial hypercholesterolemia or elevated Lp(a) in the absence of elevated triglycerides). Non-fasting lipid values may better predict the risk for ASCVD, since they provide a more accurate insight into the postprandial status of the patient and the influence of residual risk [31].
– Triglyceride concentration is the only parameter that changes substantially after food intake [31]**.** Given that the Friedewald’s formula is significantly inaccurate in patients with Tg>150 mg/dL, it is recommended that LDL-c is calculated using Martin/Hopkins formula [32] (Annex. Supplementary Material table 3) or that non-HDL cholesterol is calculated instead in these patients.
– Fasting is recommended if Tg≥4.5 mmol/L (≥398 mg/dL) prior to the initiation of a drug therapy that can cause severe hypertriglyceridaemia (i.e. isotretinoin) and in genetically predisposed subjects with a history of hypertriglyceridemic pancreatitis. Fasting is also advised when the laboratory request includes additional laboratory tests that require that samples are collected in fasting conditions, or when morning samples are required (i.e. fasting glucose or parameters affected by circadian rhythm).
– Fasting and non-fasting lipid profile should be considered as complementary rather than as mutually exclusive.
– Cholesterol and triglycerides are generally tested using enzymatic methods, with determinations having a variability <10 % (Annex Supplementary Material Table 2) [33]. However, within-subject variability and variability resulting from sample collection conditions (≈20% for triglycerides and ≈10% for HDL cholesterol and LDL cholesterol), make it necessary that lipid profile testing is repeated in primary prevention patients without a clear indication for immediate initiation of a lipid-lowering therapy [33].

**Table:** 

Recommendation 2: Fasting is not required for lipid profile evaluation as an initial cardiovascular risk assessment. If levels of triglycerides exceed Tg≥4.5 mmol/L (≥398 mg/dL), a second determination is recommended in fasting conditions for confirmation of results.

## Analytical considerations

### Should the analytical method be reported?

Lipid profile determination should always be performed using the same methods, and a change in the testing method should always be reported. It is necessary that physicians are aware of the laboratory method used in lipid profile testing, since interferences or misinterpretations may occur.

**Table:** 

Recommendation 3: Reporting the laboratory technique or a change of units is essential for a correct interpretation of laboratory results.

### Methods for the determination of LDL cholesterol

The method of reference for testing LDL-c involves separating lipoproteins by density-gradient ultracentrifugation, a time-consuming method that is only available in specialized laboratories. For this reason, LDL-c is often estimated by measuring total cholesterol and triglycerides (using enzymatic methods), and by direct HDL cholesterol determination. Friedewald’s is the most frequently used formula [34].

Friedewald’s formula for the estimation of LDL-c (in mg/dL)

LDL cholesterol=Total cholesterol – HDL cholesterol – triglycerides/ 5

Friedewald formula assumes the absence of chylomicrons and a specific of cholesterol/Tg ratio in VLDL-c(1/5 in mg/dL; 1/2.2 in mmol/L). In VLDL, the Tg/ cholesterol ratio progressively increases as hypertriglyceridaemia exacerbates; therefore, in patients with hypertriglyceridaemia, the formula overestimates VLDL-c and thus underestimates LDL cholesterol. The formula has an acceptable accuracy when Tg concentration is <200 mg/dL and it should not be used if Tg>400 mg/dL (Recommendation 4).

The Martin-Hopkins formula replaces number 5 in the Friedewald’s formula (c-VLDL= Tg/5) with divisors that vary according to the levels of Tg and non-HDL cholesterol of the patient (Annex. Supplementary Material Table) [32]. The Martin-Hopkins formula is more accurate than Friedewald’s when Tg>150 mg/dL, LDL-c <100 mg/dL, and especially when <70 mg/dL**.**

The Sampson formula is more complex and provides similar results to those of Martin-Hopkins for patients with Tg<400 mg/dL. As a result, the former is less frequently used. In patients with Tg>400 mg/dL, the use of formulas for the estimation of LDL-c is not recommended, due to their poor reliability**.**

Ultracentrifugation, the classical reference method for LDL-c determination, is labour intensive and is only used in very specialized laboratories. There is a direct, accurate, and widely-available measurement method. The use of this marker is recommended if Tg>400 mg/ dL or LDL<70 mg/dL, when LDL-c determination methods are more inaccurate [32].

When a direct method cannot be used to determine LDL cholesterol, the use of non-HDL cholesterol as a marker of “atherogenic” cholesterol is recommended [35]. Determination of apolipoprotein B can also be used (Apo B). Non-HDL cholesterol does not require Tg determination nor is it influenced by fasting. Moreover, it strongly correlates with levels of Apo B.

**Table:** 

Recommendation 4: The Friedewald formula is accurate in most patients with LDL-c > 100 mg/dL and Tg<150 mg/dL. The modified Martin-Hopkins formula is superior for the estimation of LDL cholesterol, especially in patients with low LDL-c concentrations<70 mg/dL, Tg concentrations 150–400 mg/dL, and in non-fasting conditions. Direct LDL-c assays should be used to determine LDL-c if Tg≥400 mg/dL.

In patients with significantly elevated Lp(a) concentrations, LDL-c estimation should be corrected using the following formula:

LDL−c corrected for Lp(a) (mg/dL)=LDL−c (mg/dL)−[Lp (a) (mg/dL)×0.30]

LDL−c corrected for Lp(a) (nmol/L)=LDL−c (nmol/L)−[Lp (a) (mg/dL)×0.0078]

Potential Lp(a) elevation should be especially considered in Subsaharian patients, patients with the nephrotic syndrome, on peritoneal dyalisis, or with a poor decrease of LDL-c after having received a lipid-lowering therapy.

## Post-analytical considerations

### Markers of “normality” and alerts

The clinical laboratory plays a crucial role in the assessment of cardiovascular risk in patients with dyslipidaemia. It is essential that specific reference values are established for the pediatric population.

Desirable values should be provided in terms of cardiovascular risk and prevention on lipid profile reports [14], [15], [16]. Table 2 shows the desirable values for the main lipid parameters established for adults by the European Society of Cardiology, Arteriosclerosis and the Spanish Society of Laboratory Medicine (2019) [17, 33, 36].

**Table 2:** Desirable lipid values in adults, according to the European Societies of Arteriosclerosis and Laboratory Medicine [17, 33, 36].

Parameter		Desirable value in adults
Serum total cholesterol		<200 mg/dL (5.17 mmol/L)
HDL cholesterol		>50 mg/dL women (>1.29 mmol/L)
		>40 mg/dL men (1.03 mmol/L)
LDL cholesterol		Recommended values based on CVR
		– Secondary prevention and very-high CVR<55 mg/dL (<1.4 mmol/L) – High CVR<70 mg/dL (<1.8 mmol/L) – Moderate CVR<100 mg/dL (<2.6 mmol/L) – Low CVR<116 mg/dL (<3 mmol/L)



Non-HDL cholesterol		Recommended values based on cardiovascular risk
		– Secondary prevention and very-high CVR<85 mg/dL (<2.2 mmol/L) – High CVR<100 mg/dL; (<2.6 mmol/L) – Moderate CVR<131 mg/dL (<3.4 mmol/L) – Secondary prevention and very-high CVR<85 mg/dL (<2.2 mmol/L)



Triglycerides		Fasting TG <150 mg/dL (<1.69 mmol/L)
		(Non-fasting TG <175 mg/dL) (<1.97 mmol/L)
Apolipoprotein B		Recommended values based on CVR
		– Secondary prevention and very-high CVR <65 mg/dL – High CVR <80 mg/dL – Moderate CVR <100 mg/dL (<3.4 mmol/L)
	
	
Lipoprotein (a)		<50 mg/dL (<105 nmol/L)
		<30 mg/dL in fasting conditions
		<35 mg/dL in non-fasting conditions

CVR, cardiovascular risk.

Critical values should be flagged and reported as such to the requesting physician, as shown in Table 3 **.**

**Table 3:** Recommended alerts of the information system/laboratory report.

Parameter	Critical value	
Serum total cholesterol	310 mg/dL	Patient with high cardiovascular risk
Triglycerides	>880 mg/dL	Severe hypertriglyceridaemia with risk of acute pancreatitis
Adult LDL cholesterol	>190 mg/dL	Consider heterozygous familial hypercholesterolemia
Adult LDL cholesterol	>500 mg/dL	Consider homozygous familial hypercholesterolemia
Atherogenic lipid triad	If: Tg>150 mg/dL and HDL<30 mg/dL, LDL/Apo B<1.3 or Tg/HDL>2	Lipid triad suggestive of atherogenic dyslipidemia with a very high vascular risk
Lipoprotein (a)	>120 mg/dL (260 nmol/L)^a^	Very high CV risk of atherosclerotic cardiovascular disease and aortic valve stenosis
Apolipoprotein A 1	<10 mg/dL	Consider hypoalphalipoproteinemia
Apolipoprotein B	<10 mg/dL	Consider hypo betalipoproteinemia

LDL, low density lipoproten; Apo, apolipoprotein; Tg, triglycerides; CV, cardiovascular. ^a^Estimation based on EAS/EFLM consesus document.

**Table:** 

Recommendation 5: On laboratory reports, the reference values for lipid parameters should always be referred to the cardiovascular risk of the patient, rather than to normal reference values for the general population. The use of asterisks marking values outside the population normality range is discouraged. The use of flagging systems is recommended for extreme lipid concentrations suggestive of severe dyslipidaemias. Specific values should be established for the pediatric population.

### What parameters should a basic lipid profile include?

**Figure 1:**
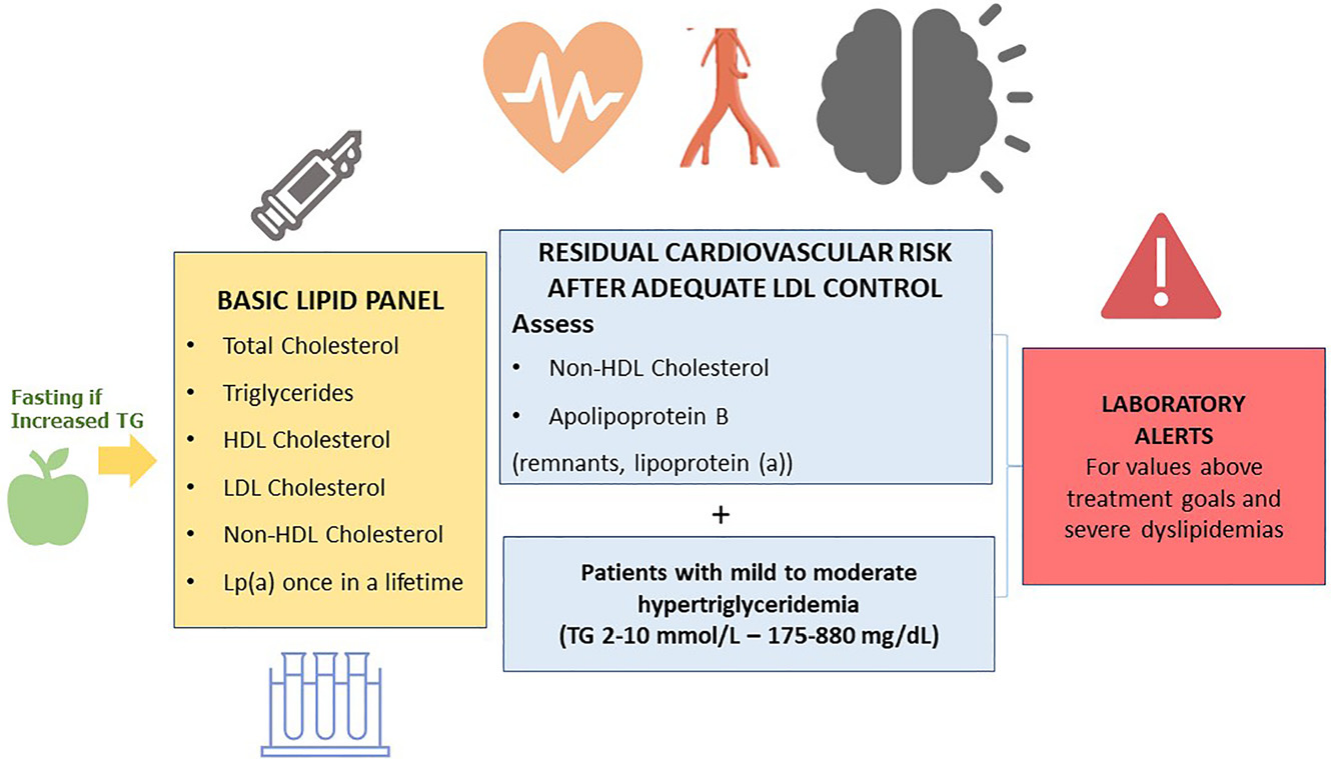
Basic recommendations for lipid profile reporting in Spanish clinical laboratories.

The basic lipid profile should include the determination of total cholesterol, HDL cholesterol, triglycerides, non-HDL cholesterol and LDL-c [9, 17, 37–39] (Figure 1).

The Consensus Statements of the European Society of Arteriosclerosis and the European Society of Laboratory Medicine also recommend the estimation of remnant particles [9–16, 36]. Elevated lipoprotein (a) confers an increased vascular risk. Therefore, it is recommended that it is measured at least once in life, since levels are substantially determined by genetic factors [9–16, 36].

In patients with Tg>400 mg/dL, direct determination of LCL cholesterol is recommended in order to obtain more reliable values [40]. If available, Apo B is a marker of special interest, since it is the best marker of the number of atherogenic lipoproteins [41]. When direct determination of LDL-c or Apo B is not available, non-HDL cholesterol can be used as an approximation.

**Table:** 

Recommendation 6: The basic lipid profile should include the determination of total cholesterol, HDL cholesterol, triglycerides, non-HDL cholesterol and LDL-c estimation. In patients with mild/moderate hypertriglyceridaemia, testing non-HDL cholesterol and Apo B is recommended for assessing residual cardiovascular risk.

### What is non-HDL cholesterol tested for?

The estimation of non-HDL cholesterol involves a simple calculation (total cholesterol – HDL cholesterol) that reflects the cholesterol of atherogenic lipoproteins. Non-HDL cholesterol strongly correlates with Apo B concentrations. Also, it is the parameter of reference in the vascular risk assessment formulas SCORE2 (Systematic Coronary Risk Evaluation) and SCOREOP (Systematic Coronary Risk Evaluation old people) [9, 42, 43]. An additional advantage of this parameter is that it is not influenced by fasting. Moreover, it can be determined in patients with Tg>400 mg/dL or be useful as a guideline in laboratories where direct LDL or Apo B determination is not available [44].

### When should apolipoprotein B used?

Apo B is an excellent predictor of cardiovascular events, since this apoprotein is found in the main atherogenic lipoproteins, namely: LDL, lipoprotein (a), VLDL-cand IDL [41], [42], [43], [44], [45]. Testing Apo B is equivalent to measuring the amount of atherogenic lipoproteins, since each one contains a single molecule of Apo B. Apo B values are not affected by fasting. The amount of lipoparticles can also be measured by MRI (magnetic resonance imaging). However, this technique is not available in routine clinical practice [46].

Apo B is especially relevant in patients with elevated triglycerides, diabetes mellitus, obesity, metabolic syndrome, or very low LDL cholesterol. In these cases, measurement or estimation of LDL-c may be inaccurate and not consider the atherogenic component of other lipoproteins.

Apo B test is rarely included in standard lipid profiles or ASCVD risk assessment tests. Monogenic disorders such as familial hypercholesterolemia (FH) can be easily recognized using a standard panel of lipids. In these cases, measuring Apo B is not necessary (Annex. Supplementary Material, Table 4) [47]. On another note, Apo B concentration helps classify severe dyslipidaemias, such as combined familial hyperlipidaemia and familial dysbetalipoproteinemia [48] (Annex. Supplementary Material. Figure).

**Table:** 

Recommendation 7: Testing Apo B is recommended for assessing vascular risk; classify dyslipidaemias and characterize particle size. It is also preferable to non-HDL cholesterol testing in patients with mild-to-moderate hypertriglyceridaemia (175–880 mg/dL), diabetes, obesity, metabolic syndrome, or very low LDL-c (<70 mg/dL).

### When should lipoprotein (a) be determined?

Testing Lp (a) is recommended at least once in life to estimate vascular risk [9, 49], [50], [51], [52]. This determination is especially relevant in patients with early-onset cardiovascular disease, familial hypercholesterolemia, poor response to statin therapy, aortic stenosis or recurrent ischemic events, and in relatives to patients with elevated Lp (a). The cardiovascular risk of patients with very elevated Lp (a) (>180 mg/dL/ >430 nmol/L) is similar to that of patients with heterozygous familial hypercholesterolemia [53, 54]. One of the challenges of Lp(a) determination is the variability of results across the different detection techniques. Another disadvantage is the unavailability of a direct equivalence between values reported in mg/dL and in nmol/L, according to the different apoprotein (a) isoforms.

Lp (a) should only be measured once in life, given that it is substantially determined by genetics and there are no specific drug therapies available as yet. Exceptions to this rule include transition to menopause, pregnancy, use of oral contraceptives, chronic kidney disease/ nephrotic syndrome, or when a specific treatment is used to reduce Lp (a) or modulate the recommended therapeutic options, such as PCSK9 inhibitors [55].

**Table:** 

Recommendation 8: Lp (a) should be determined only once in life, except when its levels may be affected by significant changes, as the development of nephrotic syndrome or the use of a therapy to reduce Lp (a). The most appropriate units of measurement are nmol/L (Annex. Supplementary Material. Comment).

### Should inflammation be assessed in patients with arteriosclerosis?

Chronic inflammatory processes are associated with an increased cardiovascular risk, regardless of the risk attributable to conventional risk factors [56]. High-sensitivity C-reactive protein is the analytical parameter most frequently used as a maker of low-intensity inflammation. It has a high variability, and there is no defined consensus on the values that should be considered ‘elevated’ for the estimation of vascular risk assessment [17].

### Innovations in the diagnosis of dyslipidaemias: parameters for an e-consultation

For an e-consultation to be rapid and effective, the basic parameters to be included for the diagnosis of dyslipidaemias are shown in Table 4.

**Table 4:** Reference data required for assessing cardiovascular risks in an e-consultation.

(1) Age, sex, BMI and waist circumference of the patient
(2) Short summary of familial and personal medical history
(3) Risk factors: Tobacco use, alcohol use (quantified),
(4) Short summary of the lipid history and previous lipid-lowering treatments
(5) Full outline of patient treatment
(6) Possible side effects of lipid-lowering therapy.
(7) Current basic lipid profile) Total cholesterol, LDL cholesterol, HDL cholesterol, Non-HDL cholesterol and triglycerides
(8) Active problem
(9) Availability of personal or familial genetic studies.
(10) In case familial hypercholesterolemia is suspected, Dutch lipid clinic network score (DLCN)/WHO (47)
(11) On case of suspicion of hypertriglyceridemia: Moulin score for the diagnosis of familial quilomicronemia (57).

BMI, body mass index; LDL, low density lipoprotein; HDL, high density lipoprotein; WHO, World Health Organization.

## Supplementary Material

Supplementary Material
