# Supplementary material for: Consensus document for lipid profile testing and reporting in Spanish clinical laboratories: what parameters should a basic lipid profile include?
Source: Adv Lab Med. 2023 Jun 5;4(2):138–46. doi: 10.1515/almed-2023-0047 (PMC10701497; doi:10.1515/almed-2023-0047)
Supplement: Supplementary file 1 — Supplementary Material [file j_almed-2023-0047_suppl_001.docx]

Appendix

**TABLE 1 A : Causes of secondary hypercholesterolaemia ^20^**

| **Clinical conditions** | Hypothyroidism | Gaucher’s disease | Nephrotic syndrome |
| --- | --- | --- | --- |
|  | Hypopituitarism | Klinefelter’s syndrome | haemolytic uremic syndrome |
|  | Diabetes mellitus | Tay-Sachs disease | Renal transplantation |
|  | Hyperparathyroidism | Nieman-Pics disease | Chronic kidney disease |
|  | Cushing’s Syndrome | Acute intermittent porphyria | Nephrosis |
|  | Hepatitis | Monoclonal gammopathy | Anorexia nervosa |
|  | HIV infection | Dysglobulinaemia | Hepatocarcinoma |
|  | Benign recurrent intrahepatic cholestasis | Obstructive liver disease | Glycogen storage disease |
|  | Congenital biliary atresia | Autoimmune disorders | Idiopathic hypercalcemia |
|  | Alagille Syndrome | Pregnancy / Breast feeding | Systemic lupus erythematosus |
|  | Polycystic ovary syndrome | Obstructive Pulmonary disease | Immunoglobulin-lipoprotein complex disorders |
|  | | | |
| **Drugs** | Anabolic steroids | Glucocorticosteroids | Thiazolidinediones |
|  | Danazole | Amiodarone | Diuretics |
|  | Isotretinoin | immunosuppressants | SGLT2 inhibitors |
|  | Oral contraceptives | Retinoids | Beta blockers |
|  | Clopidogrel | Carbamazepine | Ranitidine |
|  | Phenobarbital | Phenitoin | Famotidine |
|  | Protease inhibitors | Long-chain omega-3 fatty acids | Docosahexaenoic acid |

| **Table 1 B. Drugs That May Cause Dyslipidaemias** 21 | | | |
| --- | --- | --- | --- |
|  | **LDL Cholesterol** | **Triglycerides** | **HDL Cholesterol** |
| ***Cardiovascular /Endocrine*** | | | |
| Amiodarone | ↑Variable | ↔ | ↔ |
| *β*-Blockers^***^ | ↔ | ↑10-40% | ↓5-20% |
| Loop diuretics | ↑5-10% | ↑5-10% | ↔ |
| Thiazide diuretics (high dose) | ↑5-10% | ↑5-15% | ↔ |
| Sodium-glucose co-transporter 2 (SGLT2) inhibitors | ↑3-8% | ↔↓ | ↑Variable |
| ***Steroid Hormones/Anabolic Steroids*** | | | |
| Estrogen | ↓7-20% | ↑40% | ↑5-20% |
| Selective progestins | ↑Variable | ↓Variable | ↓15-40% |
| Selective estrogen receptor modulators | ↓10-20% | ↑0-30* | ↔ |
| Danazol | ↑10-40% | ↔ | ↓50% |
| Anabolic steroids | ↑20% | ↔ | ↓20-70% |
| Corticosteroids | ↑Variable | ↑Variable | ↔ |
| ***Antiviral Therapy*** | | | |
| Protease inhibitors | ↑15-30% | ↑15-200% | ↔ |
| Direct-acting antivirals | ↑12-27% | ↔ | ↑14-20% |
| ***Immunosuppressants*** | | | |
| Cyclosporine and tacrolimus | ↑0-50% | ↑0-70% | ↑0-90% |
| Corticosteroids | ↑Variable | ↑Variable | ↔ |
| ***Centrally- Acting Medications*** | | | |
| First-generation antipsychotics | ↔ | ↑22% | ↓20% |
| Second-generation antipsychotics | ↔ | ↑20-50% | ↔ |
| Anticonvulsants | ↑Variable | ↔ | ↑Variable |
| ***Other Medications*** | | | |
| Retinoids | ↑15% | ↑35-100% | ↔** |
| Growth Hormone | ↑10-25% | ↔ | ↔↑7% |
| ABBREVIATIONS: LDL, low-density lipoprotein; HDL, high-density lipoprotein. *Raloxifene has not been shown to increase triglyceride levels, while reported increases of up to 30% have been reported with the use of tamoxifen**Data remains conflicting and some evidence shows a decrease, no effect, or increase***Varies based on individual drug. | | | |

| **Table 2. Sources of analytical variability ^18^** | | | | |
| --- | --- | --- | --- | --- |
| **Measurement** | **Method** | **Analytes** | **Sources of Variability** | **Bias (%)* Precision (%CV) Total Error (%)** |
| Cholesterol (TC) | Enzymatic | Esterified and |  | 1.4 |
|  |  | unesterified cholesterol |  | 2.6 |
|  |  |  |  | 5.7 |
| Triglycerides | Enzymatic | Triglycerides, | (a) time and composition of | 0.4 |
| (TG) |  | diglycerides, | last meal 1 , (b) time and | 3.8 |
|  |  | monoglycerides, free | composition of last | 6.7 |
|  |  | glycerol | beverage 1 , (c) glycerol |  |
|  |  |  | kinase deficiency 2 (1/1500 in |  |
|  |  |  | the US) |  |
| High-density | Precipitation +En |  | (a) storage temperature | 3.4 |
| lipoprotein | zymatic |  | during transportation prior to | 5.3 |
| cholesterol |  |  | analysis 1 , (b) precipitation | 12.2 |
| (HDL-C, direct |  |  | agent 3 , (c) time and |  |
| HDL-C) |  |  | composition of last meal 1 , (d) |  |
|  |  |  | high TG levels 2 , (e) high |  |
|  |  |  | Lp(a) levels 2 |  |
| Friedewald- | Calculation | Calc-LDL-C = [TC] – | (a) time and composition of | 2.0 |
| calculated low- |  | [HDL-C] – 0.20*[TG] | last meal 1 , (b) time and | 4.8 |
| density |  |  | composition of last | 9.9 |
| lipoprotein |  |  | beverage 1 , (c) high TG |  |
| cholesterol |  |  | levels 2 , (d) high Lp(a) |  |
| (Calc-LDL-C) |  |  | levels 2 |  |
| Directly- | Precipitation +En |  | a) time and composition of | 6.1 |
| measured low- | zymatic |  | last meal 1 , (b) time and | 5.1 |
| density |  |  | composition of last | 14.6 |
| lipoprotein |  |  | beverage 1 , (d) TG levels 2 , |  |
| cholesterol |  |  | (e) HDL-C levels 2 , (f) |  |
| (dLDL-C) |  |  | elevated Lp(a) levels 2 |  |
| Non-HDL-C | Calculation | [TC]-[HDL-C] | Same as for TC and HDL-C | 1.2 |
|  |  |  |  | 3.3 |
|  |  |  |  | 6.7 |

TABL3 3.

**Median Triglycerides to Very Low-Density Lipoprotein Cholesterol ratio by Non–High- Density Lipoprotein Cholesterol and Triglyceride Strata. Martin-Simpson formula. Adapted from reference ^33^**

Non-HDL Cholesterol, mg/dL

| Triglycerides, mg/dL | <100 | 100-129 | 130-159 | 160-189 | 190-219 | ≥220 |
| --- | --- | --- | --- | --- | --- | --- |
| 7-49 | 3.5 | 3.4 | 3.3 | 3.3 | 3.2 | 3.1 |
| 50-56 | 4.0 | 3.9 | 3.7 | 3.6 | 3.6 | 3.4 |
| 57-61 | 4.3 | 4.1 | 4.0 | 3.9 | 3.8 | 3.6 |
| 62-66 | 4.5 | 4.3 | 4.1 | 4.0 | 3.9 | 3.9 |
| 67-71 | 4.7 | 4.4 | 4.3 | 4.2 | 4.1 | 3.9 |
| 72-75 | 4.8 | 4.6 | 4.4 | 4.2 | 4.2 | 4.1 |
| 76-79 | 4.9 | 4.6 | 4.5 | 4.3 | 4.3 | 4.2 |
| 80-83 | 5.0 | 4.8 | 4.6 | 4.4 | 4.3 | 4.2 |
| 84-87 | 5.1 | 4.8 | 4.6 | 4.5 | 4.4 | 4.3 |
| 88-92 | 5.2 | 4.9 | 4.7 | 4.6 | 4.4 | 4.3 |
| 93-96 | 5.3 | 5.0 | 4.8 | 4.7 | 4.5 | 4.4 |
| 97-100 | 5.4 | 5.1 | 4.8 | 4.7 | 4.5 | 4.3 |
| 101-105 | 5.5 | 5.2 | 5.0 | 4.7 | 4.6 | 4.5 |
| 106-110 | 5.6 | 5.3 | 5.0 | 4.8 | 4.6 | 4.5 |
| 111-115 | 5.7 | 5.4 | 5.1 | 4.9 | 4.7 | 4.5 |
| 116-120 | 5.8 | 5.5 | 5.2 | 5.0 | 4.8 | 4.6 |
| 121-126 | 6.0 | 5.5 | 5.3 | 5.0 | 4.8 | 4.6 |
| 127-132 | 6.1 | 5.7 | 5.3 | 5.1 | 4.9 | 4.7 |
| 133-138 | 6.2 | 5.8 | 5.4 | 5.2 | 5.0 | 4.7 |
| 139-146 | 6.3 | 5.9 | 5.6 | 5.3 | 5.0 | 4.8 |
| 147-154 | 6.5 | 6.0 | 5.7 | 5.4 | 5.1 | 4.8 |
| 155-163 | 6.7 | 6.2 | 5.8 | 5.4 | 5.2 | 4.9 |
| 164-173 | 6.8 | 6.3 | 5.9 | 5.5 | 5.3 | 5.0 |
| 174-185 | 7.0 | 6.5 | 6.0 | 5.7 | 5.4 | 5.1 |
| 186-201 | 7.3 | 6.7 | 6.2 | 5.8 | 5.5 | 5.2 |
| 202-220 | 7.6 | 6.9 | 6.4 | 6.0 | 5.6 | 5.3 |
| 221-247 | 8.0 | 7.2 | 6.6 | 6.2 | 5.9 | 5.4 |
| 248-292 | 8.5 | 7.6 | 7.0 | 6.5 | 6.1 | 5.6 |
| 293-399 | 9.5 | 8.3 | 7.5 | 7.0 | 6.5 | 5.9 |
| ≥ 400 | 11.9 | 10.0 | 8.8 | 8.1 | 7.5 | 6.7 |

TABLE 4 Diagnostic Score table for familial hypercholesterolaemia (designed by the Dutch Lipid Clinic Network ) ^47^


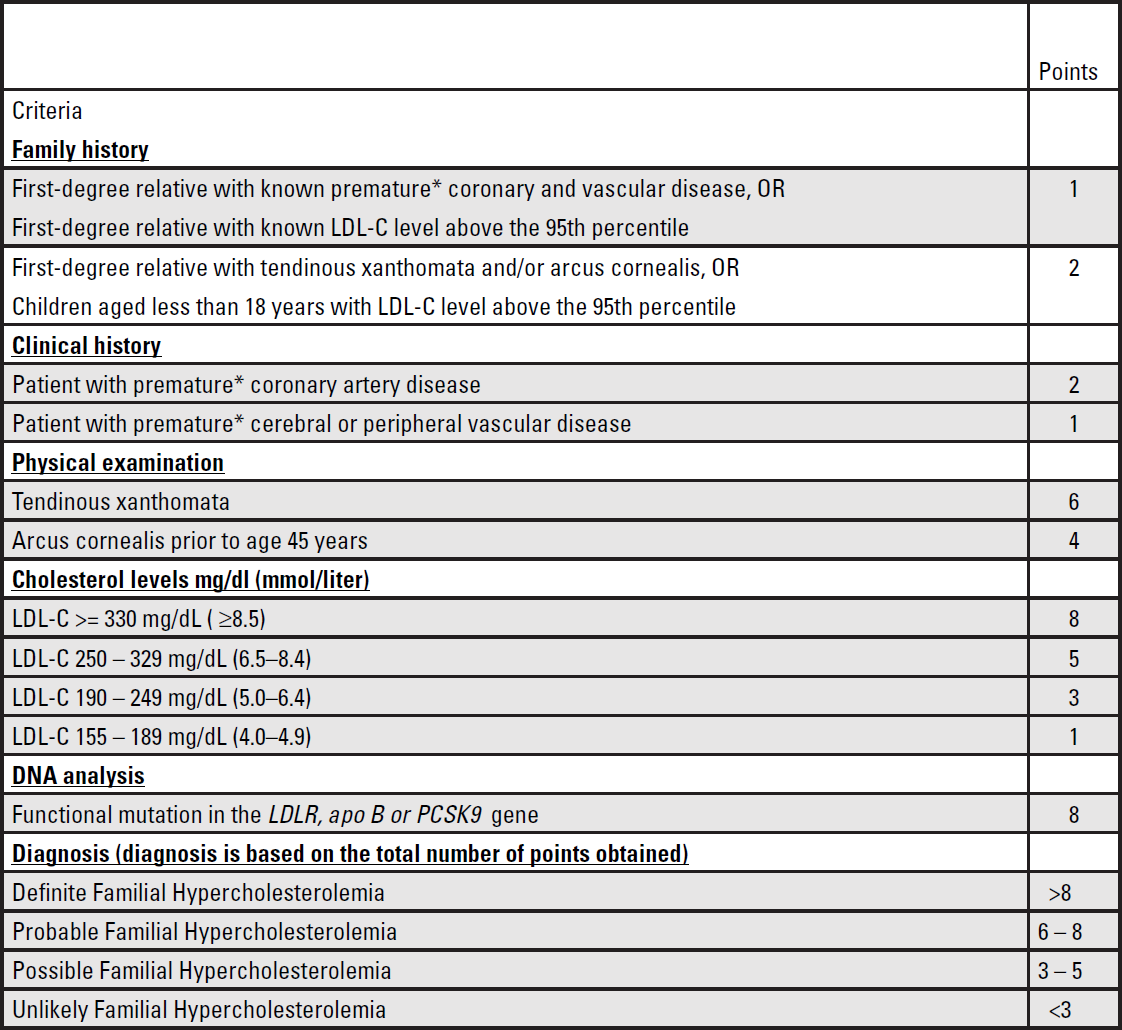


TABL 5. Moulin score for the diagnosis of Familial Chyilomicronaemia Syndrome ^57^


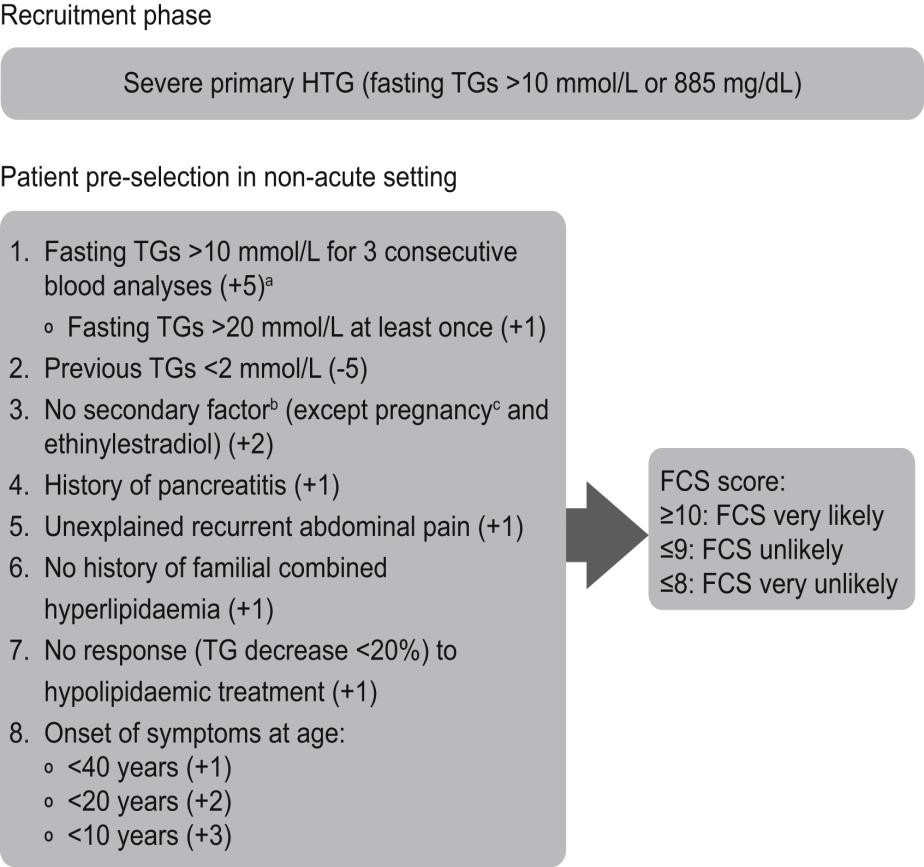


Numbers in parentheses = weighting given to the presence of each item. FCS score = the sum of all items present. FCS, familial chylomicronaemia syndrome; HTG, hypertriglyceridaemia; TG, triglyceride. a Plasma TG concentration measured at least one month apart. Eruptive xanthoma may be used as a surrogate for high TG levels (rare). b Secondary factors include alcohol, diabetes, metabolic syndrome, hypothyroidism, corticotherapy and additional drugs]. c If diagnosis is made during pregnancy, a second assessment is necessary to confirm diagnosis post-partum.

FIGURE. Simplified algorithm for the diagnosis of dysbetalipoproteinemia ^45^


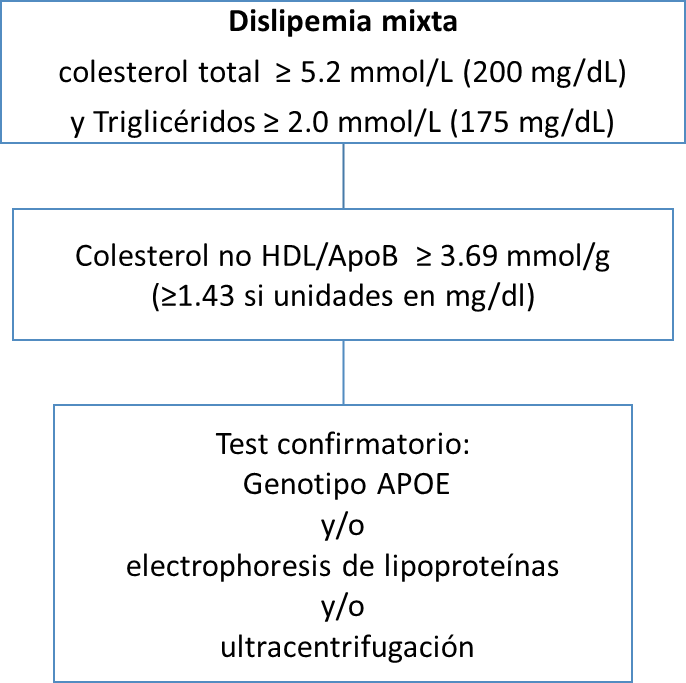


Confirmatory test: Apo E Genotype and/or

Protein electrophoresis and/ or ultracentrifugation

Non-HDL cholesterol / Apo B ≥3.69 mmol/g (≥1.43 if units in mg/dL)

**Mixed dyslipidaemia**

Total cholesterol ≥ 5.2 mmol/L (200 mg/dl) And Triglycerides ≥ 2.0 mmol/L (175 mg/dL)

COMMENT:

Controversies in units of determination Lp(a):

In clinical practice, the determination of Lp(a) levels is generally carried out by means of immunoassays that use polyclonal antibodies against apo(a). Results are usually expressed as the total mass of the Lp(a) particle in (mg/dL), which includes both the mass of apo(a) and the other components of the particle (apoB, cholesterol, cholesterol esters), phospholipids, triglycerides, and carbohydrates) or as a molar concentration of apo(a) in nmoL/L. From the metrological point of view, the expression of results as total mass (mg/dL) has important limitations due to the great variability that Lp(a) presents in its composition and the lack of a reference material to which to refer the results. calipers. On the contrary, the expression of results in nmol/L of apo(a) reflects more precisely the plasmatic concentration of Lp(a) particles and also allows comparison between different methods by having a reference material ( WHO/IFCC SRM- 2B) to which calibrators can be referenced.

Conversion between units from nmol/L to mg/dL, or vice versa, is not recommended, as all conversion factors are intrinsically dependent on isoforms.
